# Structural Patterns under X-Rays: Is SNOMED CT Growing Straight?

**DOI:** 10.1371/journal.pone.0165619

**Published:** 2016-11-03

**Authors:** Pablo López-García, Stefan Schulz

**Affiliations:** Institute for Medical Informatics, Statistics and Documentation, Medical University of Graz, Graz, Austria; Stanford University Medical Center, UNITED STATES

## Abstract

Unprincipled modeling decisions in large-domain ontologies, such as SNOMED CT, are problematic and might act as a barrier for their quality assurance and successful use in electronic health records. Most previous work has focused on clustering problematic concepts, which is helpful for quality control but faces difficulties in pinpointing the origin of those modeling problems. In this study, we examined the underlying structural patterns in SNOMED CT’s data model as such patterns directly reflect the modeling strategies of editors. Our results showed that 92% of all structural patterns found accumulated in the *Procedure* and *Clinical finding* sub-hierarchies, and pattern reuse was low; over 30% of patterns were only used once. A qualitative analysis of a sample of 50 such singleton patterns revealed modeling problems, including redundancy, omission, and inconsistency. The problems detected in the sample suggest that the analysis of structural patterns is a valuable technique for revealing problematic areas of SNOMED CT and modeling the styles of terminology editors. Furthermore, the patterns that describe the modeling of a large number of concepts could provide insights for template creation and refinement in SNOMED CT.

## Introduction

SNOMED CT is a comprehensive multilingual clinical terminology designed to support the consistent and processable representation of clinical content in electronic health records [1]. With over two million biomedical terms in different languages, SNOMED CT has been extensively studied in terms of validity, coverage, and reliability for coding, showing very promising results [2–4].

In contrast with other terminologies, SNOMED CT is based on a solid ontological framework that uses formal reasoning in its content production and maintenance. In SNOMED CT, terms are organized hierarchically in over 300 000 representational units, called SNOMED CT concepts, which are described by formal axioms conforming to the EL++ description logics standard [5]. In this sense, SNOMED CT is unique among the currently used clinical terminologies. Its sheer size means that formal or automated methods are required for quality assurance and that new methodologies for examining it are needed, a fact that has been acknowledged since the preliminary stages of SNOMED CT, when lexical techniques were already being investigated [6].

Research has shown that content production and maintenance of SNOMED CT are indeed problematic. In 2007, Bodenreider et al. [7] found out that 51% of SNOMED CT concepts did not exhibit any descriptive difference between parent and child, i.e., the concepts were modeled using bare **is-a** relationships. After analyzing a small sample, the authors discovered that most descriptions of those SNOMED CT concepts were indeed incomplete. A study conducted in 2012 confirmed these findings: Mikroyannidi et al. [8] inspected three modules from SNOMED CT that were expected to instantiate similar patterns described in the SNOMED CT technical guide and found that incomplete descriptions were the cause of most regularity deviations. Since then, SNOMED CT has been significantly completed and improved by the International Health Terminology Standards Development Organization (IHTSDO) [1], but quality issues still persist.

In 2014, Dentler et al. [9] showed that 12% of SNOMED CT concepts contained redundant axioms in their descriptions, i.e., clauses that are stated in the formal description of a given concept but which can also be inferred from a parent, therefore unnecessarily complicating editing and maintenance.

In addition to incompleteness and redundancy, perhaps the most worrying issue in SNOMED CT is unprincipled modeling. Giannangelo et al. [10] identified inconsistent modeling as a barrier for the successful use of SNOMED CT in electronic health records. Jiang and Chute used formal concept analysis to audit SNOMED CT’s sub-hierarchies and found that the semantic completeness of several sub-hierarchies (e.g., *Clinical finding* and *Procedure*) was significantly different [11]. In good agreement with these observations, Rector et al. [12] examined SNOMED CT’s hierarchies in the *Core Problem List Subset of SNOMED CT* and came to the following disturbing conclusion: SNOMED CT hierarchies in their current state were not reliable, and using them might negatively affect interoperability and meaningful use. The *CORE problem list subset of SNOMED CT* is a subset of 6 346 SNOMED CT concepts that contains the terms most frequently used when annotating clinical information at a summary level in several disciplines [13].

Several approaches for systematically finding such modeling problems in SNOMED CT have been proposed. More recent lexical approaches than those of Campbell et al. [6] include that of Pacheco et al. [14], who used natural language processing techniques to find approximately 18 000 SNOMED CT concepts for which the wording suggested a connection to other concepts, but such connections were missing. In a similar way, Agrawal et al. [15] suggested that SNOMED CT concepts that are lexically similar should be structurally similar as well and clustered them into *positional similarity sets*. The manual analysis of a small random sample of positional similarity sets revealed several modeling problems.

Another approach for detecting modeling problems in SNOMED CT is to investigate its structure, an approach for which a rich body of research already exists. In 2006, Zhang et al. [16] reverse-engineered SNOMED CT’s distribution files and found 110 relationship patterns among SNOMED CT’s 19 top-level sub-hierarchies; patterns in the form of [*top-level sub-hierarchy*, **relationship type**, *top-level sub-hierarchy*], which provide a starting point to better understand SNOMED CT and its associated modeling problems as a whole.

Using abstraction networks to partition SNOMED CT has consistently been shown to be useful for auditing SNOMED CT concepts (and relationships [17]), and supporting tools have been developed for that purpose (BLUSNO [18]). In 2007, Halper et al. [19] and Wang et al. [20] partitioned SNOMED CT automatically into areas and p-areas and assessed a small sub-hierarchy (*Specimen)*, revealing problematic concepts. Their hypothesis was that rarely seen combinations of structure and semantics might reveal concepts that should be considered for revision. In fact, p-areas in the *Specimen* sub-hierarchy containing only one concept, termed *singletons*, contained a higher percentage of erroneous concepts. Further refinements in their abstraction networks, reassessments of the *Specimen* sub-hierarchy, and improvements for scalability strengthened this hypothesis [21–25]. The results of all previous studies are in good agreement with Mikroyannidi et al. [8], who also showed that parts of an ontology that do not follow an explicit modeling pattern tend to have more potential defects.

In this study, we build on previous structural research on SNOMED CT [16, 19, 21] to further investigate modeling problems in SNOMED CT. In contrast with previous research, we introduce and analyze *structural patterns* (see the Materials and Methods section) because structural patterns reflect how individual concepts were described by the editors in the stated version of SNOMED CT. Optimally, many concepts should share the same structural pattern, especially those within the same sub-hierarchy and at the same depth level.

Our hypothesis is that the areas of SNOMED CT that accumulate rarely used structural patterns—in particular *singleton patterns*, i.e., structural patterns that only occur once—should reveal important modeling problems in SNOMED CT, as was the case with *singleton p-areas* that revealed a higher percentage of erroneous concepts in the *Specimen* sub-hierarchy [19]. The analysis of structural patterns applied to a large number of concepts could not only pinpoint where modeling problems accumulate in SNOMED CT but also reveal valuable information about the modeling styles of terminology editors.

## Materials and Methods

SNOMED CT is divided into 19 sub-hierarchies headed by top-level concepts (e.g., *Body structure*, *Clinical finding*, *Organism*, *Substance*). The sub-hierarchy to which a SNOMED CT concept belongs can be determined by following its taxonomical (**is-a)** links to the top.

In SNOMED CT, concepts are logically described using triples in the form [*Concept*, **Relationship**, *Concept*]. Relationships can be of two types: (i) **is-a** relationships, which taxonomically connect concepts to more general concepts from the same sub-hierarchy and correspond to OWL subClassOf axioms, and (ii) **attribute** relationships, which introduce necessary criteria for further definition or characterization of concepts and observe the SNOMED CT concept model [26] (e.g., **Finding site**, connecting clinical findings with their anatomical sites; **Causative agent**, connecting disorders with associated pathogens; and **Active ingredient,** connecting drug products with their constituent chemicals). SNOMED CT triples encode formal axioms that conform to the EL++ description logics standard and correspond to (i) subclass axioms (**is-a** relationships) and to (ii) description logic restrictions using object properties in an existentially qualified role (**attribute** relationships) [27]. The latter can be used to infer additional subclass axioms using a description logic classifier.

As a result, SNOMED CT exists in two versions: the *stated* version and the *inferred* version. The stated version is the version edited and maintained by the SNOMED CT editors [26], whereas the inferred version also contains all logically inferable direct **is-a** links as computed by a description logic classifier. Because of the relatively weak EL++ description logic and the fact that SNOMED CT does not have disjoint class axioms, hierarchical relationships that exist in the stated version cannot be found inconsistent in the inferred version.

For example, in the stated version of SNOMED CT, *Neoplasm of kidney* is fully defined by the editors as a *Neoplastic disease* that has **Finding site** *Kidney structure* and **Associated morphology** *Neoplasm*. Because it can be logically inferred that *Neoplasm of kidney* is also a *Kidney disease*, this additional **is-a** relationship is present in the inferred version. As our goal was to study modeling problems in SNOMED CT and their underlying causes, the *stated version* (which reflects how concepts were modeled by editors) was used in this study. The patterns of how relationships have been stated by terminology editors determined what could be inferred. This is the key difference between this paper and the previous studies that were mainly focused on the inferred version. Table 1 provides an example of how SNOMED CT triples and description logic terminology relate.

**Table 1:** Synopsis of SNOMED CT's relational release format with its underlying semantics in description logics (OWL-EL) and text for the concept “Neoplasm of kidney” in the stated version. Throughout the paper, we use the OWL Manchester syntax [28], *italics* for concept (class) names and boldface for relationship names (SNOMED CT linkage concepts / OWL subClassOf and object properties).

SNOMED CT triple	Corresponding OWL axiom	Textual description
*[Neoplasm of kidney*, **Is-a,** *Neoplastic disease]*	*Neoplasm of kidney* **subClassOf** *Neoplastic disease*	Every kidney neoplasm is a neoplastic disease
*[Neoplasm of kidney*, **Finding site**, *Kidney structure]*	*Neoplasm of kidney* **subClassOf** (**Finding site** some *Kidney structure*)	Every kidney neoplasm is located at some anatomical site of the type kidney structure (i.e., kidney or any of its parts) [29]
*[Neoplasm of kidney*, **Associated morphology**, *Neoplasm]*	*Neoplasm of kidney* **subClassOf** (**Associated morphology** some *Neoplasm*)	Every kidney neoplasm has an associated morphology of the type neoplasm (i.e., neoplasm or any of its descendants)

Based on the findings of Zhang et al. [16], we built on their idea of derived relationship patterns by refining them to study stated relationships at the SNOMED CT concept level and not just among top-level sub-hierarchies. The sub-hierarchy containing metadata concepts (*SNOMED CT model component*) was not considered in this study.

We define a *structural pattern* for a concept *C* as the set of *(****Relationship***, *Sub-hierarchy)* pairs obtained when replacing the right-hand of *C*’s stated relationships with its associated top-level concept and removing duplicates. See Table 2 for an example of structural pattern calculation and our notation.

**Table 2:** Stated relationships and structural pattern for the SNOMED CT concept “*Fine needle biopsy of kidney (procedure)*” in the stated version. See Table 3 for sub-hierarchy abbreviations.

Stated Relationships	Structural Pattern
**Is-a** *Fine needle biopsy*	**Is-a** *Procedure*
**Is-a** *Kidney biopsy*	**Is-a***Procedure* *(duplicate)*]
	Notation: [**Is-a**-*PR*]
**Method** *Fine needle aspiration biopsy*	**Method** *Qualifier value*
**Procedure site—Direct** *Kidney structure*	**Procedure site—Direct** *Body structure*
**Using device** *Fine biopsy needle*, *device*	**Using device** *Physical object*
	Notation: [**Method**-*QV*, **Procedure site—Direct**-*BS*, **Using device**-*PO*]

Although each specific set of stated relationships is unique, it is expected that its associated structural pattern is not. For example, a different super-concept in the **is-a** taxonomy, or a different **Method** or **Procedure site** relationship for a given concept, will not alter its structural pattern. A useful analogy is to think of each structural pattern as a template that was filled by a SNOMED CT editor when adding the concept. The patterns identified in this study will be useful for the creation of templates for future SNOMED CT authoring.

**Fig 1:**
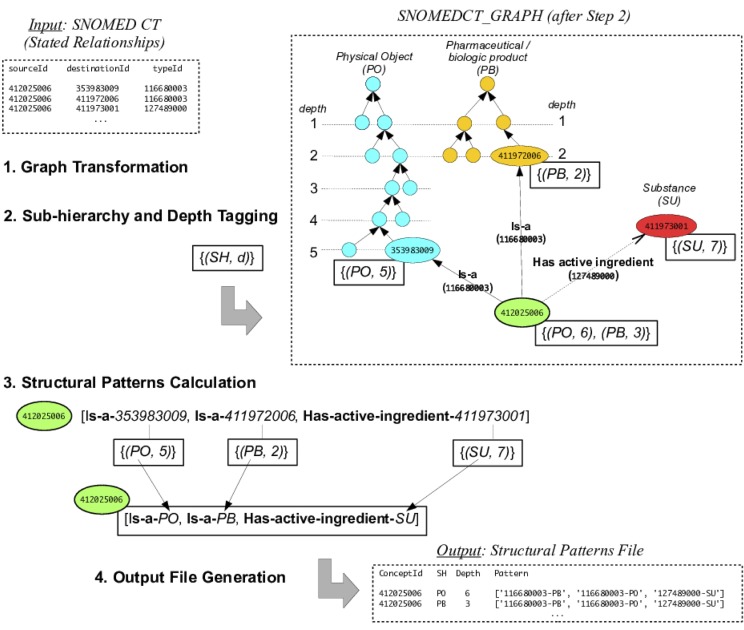
Steps followed to tag SNOMED CT concepts by top-level concept, depth, and structural pattern. Note that some SNOMED CT concepts belonged to several sub-hierarchies, as shown in this example (*Thrombin embedded bandage*, ID 412025006) and needed to be tagged twice. In SNOMED CT’s stated relationships file, *sourceId* denotes the concept to be defined, *destinationId* the target concept, and **typeId** the relationship, i.e., [*SourceId*, **typeId**, *destinationId*] triples using our notation. See Table 3 for abbreviations.

To investigate these structural patterns in SNOMED CT, the *stated relationships (snapshot)* file of the January 2015 SNOMED CT International Release (RF2) [30] was used as input. This is the *stated* version of SNOMED CT that directly reflects how concepts were modeled by SNOMED CT editors, as opposed to the inferred version normally used in applications, which also contains all description logic inferences. This file uses numerical identifiers for both SNOMED CT concepts and relationships, but both can be easily reassembled into human-readable text using other SNOMED CT distribution files. Our approach was the following:

1. **Graph Transformation.** The stated relationships file was transformed into a directed graph, *SNOMEDCT_GRAPH*, using Python’s NetworkX library [31]. Concepts were stored as nodes, and relationships as edges. Only active concepts and active relationships were considered.
2. **Sub-hierarchy and Depth Tagging.** The first step was to associate *(SH*, *d)* pairs to every concept *C* in *SNOMEDCT_GRAPH*, where *SH* represented the sub-hierarchy to which *C* belonged and *d* the deepest level where *C* was found in that sub-hierarchy. All 18 SNOMED CT sub-hierarchies under consideration were traversed from top to bottom (from less to more specific concepts), and concepts were tagged by the top-level concept and depth. This extra information was added to *SNOMEDCT_GRAPH*. Note that in SNOMED CT, concepts can belong to several sub-hierarchies and therefore be tagged more than once (see Fig 1, where the concept *Thrombin embedded bandage* (ID 412025006) is tagged with both *(PO*, *6)* and *(PB*, *3)*).
3. **Structural Patterns Calculation.** As shown in Table 2, the goal was to extract a set of *(****Relationship***, *Sub-hierarchy)* pairs for each concept *C* in SNOMED CT. Using our graph-traversal approach, for each node *N* in the *SNOMEDCT_GRAPH* directed graph (which represented a SNOMED CT concept *C*), all successor *(edge*, *node)* pairs of *N* (representing right-hand *(****Relationship***, *Concept)* pairs from the stated relationships) were traversed. Because each concept in the graph had already been tagged with sub-hierarchy information (Step 2), successor concepts were substituted by their sub-hierarchy tag, now conforming *(****Relationship***, *Sub-hierarchy)* pairs for *C*. After eliminating duplicates, (**Relationship**, *Sub-hierarchy*) pairs were collected, sorted internally (to be compared and grouped later), and assembled into a structural pattern, which was also added as a tag to node *N*. Fig 1 shows all steps graphically.
4. **Output File Generation.** The graph was finally traversed and stored in tabular form in a data file containing SNOMED CT concept identifiers (*concept_id*) and their corresponding top-level concepts (*sh*), *depths*, and *patterns*, as tagged in the previous steps. As shown in Fig 1, structural patterns were represented as a list of pairs consisting of the SNOMED CT relationship identifier plus the top-level concept tag (a two-letter acronym).

The structural patterns data file was finally processed with the R statistics package [32] to study the structural patterns as follows: (i) breadthwise, to determine how structural patterns were distributed among SNOMED CT sub-hierarchies; (ii) depthwise, to determine how structural patterns were distributed depending on the sub-hierarchy depth; and (iii) by usage, to identify rarely used patterns, particularly those that were used by one SNOMED CT concept only, which we termed *singleton patterns*, using a parallel terminology to Halper et al. [19] and Wang et al. [20].

To evaluate our hypothesis (that areas of SNOMED CT accumulating rarely used structural patterns, in particular, *singleton patterns*, should reveal important modeling problems in SNOMED CT), we examined a sample of the singleton patterns found and looked for modeling omissions, redundancies, or inconsistencies in a sample of 50 concepts stratified by sub-hierarchy and depth.

## Results

After analyzing our data file, we found 852 structural patterns in SNOMED CT’s 300 000 concepts.

## Distribution of Structural Patterns

Our first goal was to inspect structural patterns breadthwise. Table 3 shows SNOMED CT’s 18 top-level sub-hierarchies and their corresponding abbreviations, and how structural patterns and concepts belonging to them were distributed. Note that the sub-hierarchy containing metadata (*SNOMED CT Model Component*) was not considered.

**Table 3:** Distribution of structural patterns, attributes, and concepts among SNOMED CT’s 18 top-level sub-hierarchies.

	SNOMED CT Top-level Sub-hierarchy	Structural Patterns	Attributes	Concepts
PR	Procedure	587	29	54 063
CF	Clinical finding	195	16	102 217
SN	Specimen	28	5	1 455
SI	Situation with explicit context	15	7	3 762
EV	Event	8	5	3 665
PB	Pharmaceutical / biologic product^a^	6	2	16 965
PO	Physical object^a^	3	2	14 288
BS	Body structure	2	2	30 774
EG	Environment or geographical location	1	0	1 814
OE	Observable entity	1	0	8 372
OR	Organism	1	0	32 912
PF	Physical force	1	0	170
QV	Qualifier value	1	0	9 363
RA	Record artifact	1	0	225
SO	Social context	1	0	4 710
SP	Special concept	1	0	648
ST	Staging and scales	1	0	1 325
SU	Substance	1	0	24 719
	**TOTAL**^a^	**852(**	**68**	**311 436**

^a^Eleven concepts belonged to both *Pharmaceutical/biologic product* and *Physical object* sub-hierarchies and share two structural patterns: [**Is-a**-*Pharmaceutical / biologic product*, **Is-a**-*Physical object*] and [**Is-a**-*Pharmaceutical / biologic product*, **Is-a**-*Physical object*, **Has active ingredient**-*Substance*]. Therefore, the total figures do not add up to the rows above (311 447–11 = 311 436 concepts, and 854–2 = 852 structural patterns).

As shown in Table 3, the distribution of structural patterns was very heterogeneous:

**Table:** 

**1.** Ten sub-hierarchies contained only one structural pattern: Environment or geographical location, Observable entity, Organism, Physical force, Qualifier value, Record artifact, Social context, Special Concept, Substance, and Staging and scales. The structural patterns found were inevitably taxonomic (**is-a** links are the minimum structural pattern), linking concepts to their own sub-hierarchy. According to the SNOMED CT concept model, no attribute relationships are foreseen for the content of these sub-hierarchies.	
**2.** One sub-hierarchy, Body structure, contained two structural patterns:	
• Body structure:	(1) [**Is-a**-Body structure]
	(2) [**Is-a-**Body structure, **Is-a**-Qualifier value]
Interestingly, no patterns with the **Part of** relationship were obtained using our method because **Part of** is not included in the stated relationships file of SNOMED CT. Partitive relationships between anatomy concepts are currently expressed using is-a relationships among Body structure concepts, following the SEP triplet modeling pattern [29].	
3. The Physical object sub-hierarchy was the only one showing three patterns:	
• Physical object:	(1) [**Is-a**-Physical object]
	(2) [**Is-a**-Physical object, **Is-a**-Pharmaceutical / biologic product]
	(3) [**Is-a**-Physical object, **Is-a**-Pharmaceutical / biologic product,
	**Has active ingredient-**Substance]
4. Procedure (PR, 587) and Clinical finding (CF, 195) alone accounted for 92% of the total patterns found. There were a small number of patterns among Specimen (SN, 28), Situation with explicit context (SI, 15), Event (EV, 8), and Pharmaceutical and biologic product (PB, 6).	

Table 3 also shows the correlation between the number of attributes and the number of patterns for each sub-hierarchy. *Procedure* had 587 patterns for 29 attributes, a much higher ratio than that found in *Clinical finding*, with 195 patterns for 16 attributes. The numbers of patterns were much smaller for hierarchies with fewer than 10 attributes. The increased number of attributes has enabled concept modeling for different subject areas within a hierarchy. However, the potential variations of attribute combination are also increased by the number of attributes.

Our results contrast strongly with the 110 patterns found by Zhang et al. [16] because they only define patterns as relationships between top-level concepts and not as collections of such relationships, as we do. For example, our structural pattern under the *Procedure* sub-hierarchy [**Is-a**–*Procedure*, **Method**–*Qualifier value*, **Procedure site**–*Body structure*, **Using device**–*Physical object*, **Using access device**–*Physical object*] was not a pattern in their study because the five individual "**Relationship**–*Top-level concept*" relationships linking *Procedure* to other top categories are considered separate patterns. Such an interpretation, however, ignores patterns at the concept level, which are the ones that reflect how SNOMED CT editors model such concepts.

**Fig 2:**
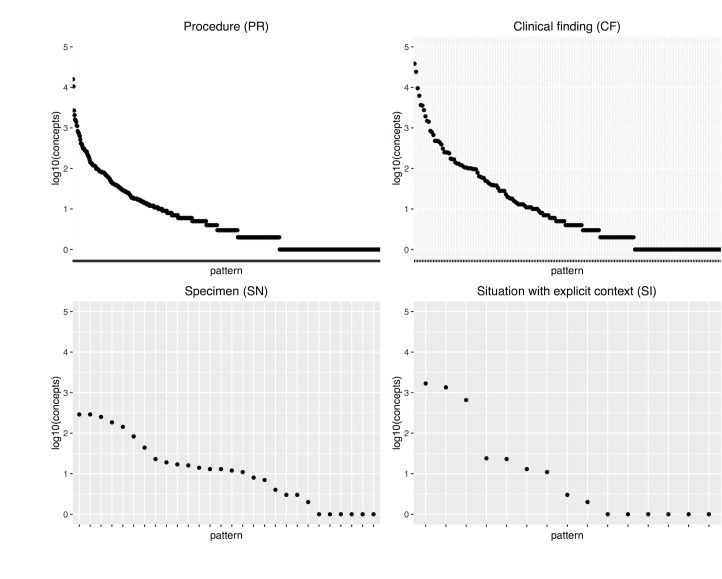
Distribution of structural patterns for the most populated top-level sub-hierarchies. The x-axis contains patterns found, the y-axis the number of concepts corresponding to that pattern (in logarithmic scale). See Table 3 for abbreviations.

Fig 2 shows the concept distribution by pattern for the most populated sub-hierarchies. The frequency of patterns in sub-hierarchies with many patterns, e.g., *Procedure* (PR) or *Clinical finding* (CF), follow a long-tailed Zipf distribution [33]. Both natural language and collections of annotations using the Gene Ontology follow this statistical pattern [34], the same phenomenon shown here.

**Fig 3:**
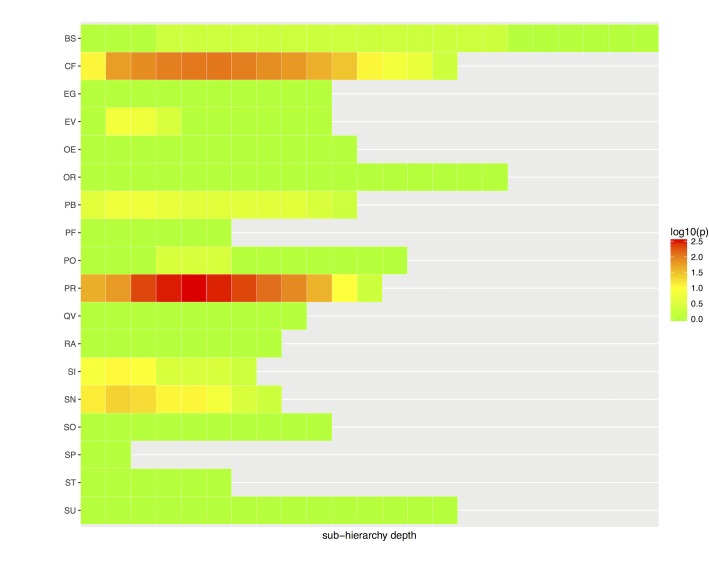
Absolute accumulation of patterns (p) per sub-hierarchy and depth. Orange and red indicate an accumulation of more than 10 patterns. The scale is logarithmic.

Our second goal was to analyze structural patterns depthwise, i.e., to investigate the influence of sub-hierarchy depth in patterns. Fig 3 shows SNOMED CT top-level sub-hierarchies and how patterns accumulated depthwise.

**Fig 4:**
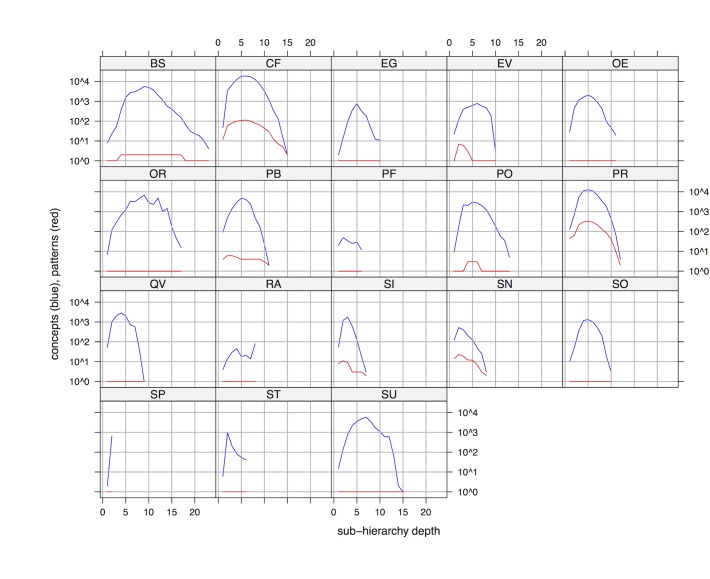
Concepts (in blue) and patterns (in red) by depth in SNOMED CT top-level sub-hierarchies. See Table 3 for abbreviations.

The most salient sub-hierarchies were *Procedure* (PR) and *Clinical finding* (CF), systematically accumulating more than 10 patterns at most depth levels. Both sub-hierarchies also showed a strong pattern variation between depth levels, with very few patterns used the deepest levels. This reduction in pattern usage is explained by the associated reduction in concept counts at the deepest levels, as shown in Fig 4.

**Fig 5:**
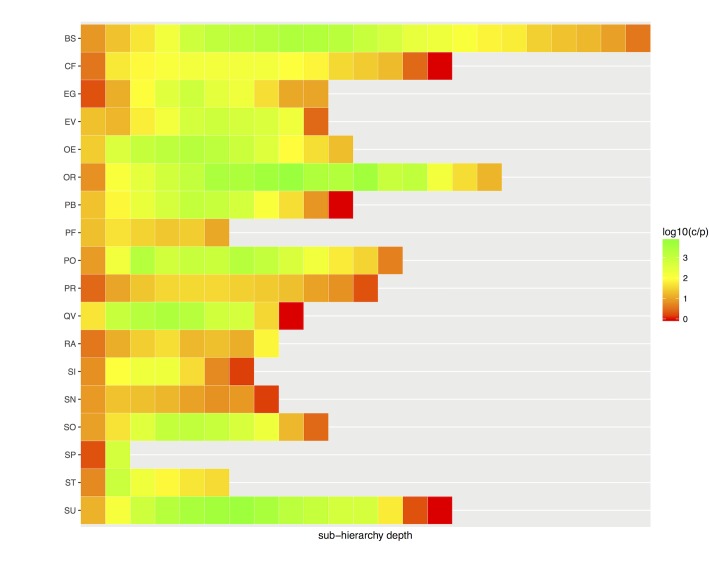
Relative usage of patterns per sub-hierarchy and depth (c/p). Reds indicate that, on average, each pattern is used by fewer than 100 concepts. The scale is logarithmic. See Table 3 for abbreviations.

In good agreement with Fig 3, the number of structural patterns in *Procedure* and *Clinical finding* were suspiciously correlated with the number of concepts, suggesting that many patterns might have been created ad hoc. As a side result when inspecting the variation in concepts by depth (Fig 4), the number of concepts dramatically decreased after depth 5 in most cases. As a last indicator for pattern usage, we investigated the average number of concepts per pattern (Fig 5).

In many sub-hierarchies and at different depths, there were many areas (marked in red) where, on average, fewer than 100 concepts corresponded to a specific structural pattern. In good agreement with previous results, underutilization of patterns (marked in red) was high on average, suggesting that many concepts in SNOMED CT have not been added following a given pattern or template, but vice versa.

## Singleton Patterns Analysis

**Fig 6:**
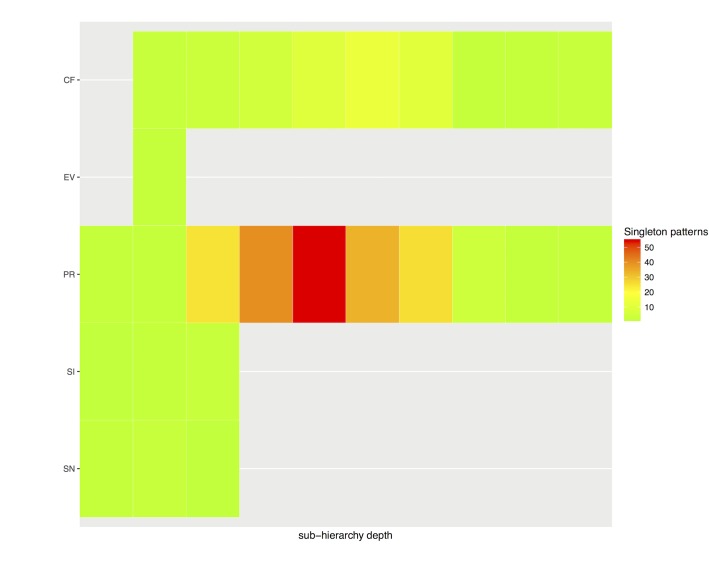
Accumulation of singleton patterns per sub-hierarchy and depth. Red indicates that over 50 patterns are accumulated. See Table 3 for abbreviations.

We identified 261 structural patterns with only one concept (192 *Procedures*, 55 *Clinical findings*, 6 *Situations with explicit context*, 6 *Specimens*, and 2 *Events*), so-called singleton patterns. Among the 852 structural patterns found, singleton patterns constituted a surprising 30%. In good agreement with the overall accumulation of structural patterns (Fig 3), *Procedure* and *Clinical finding* accumulated over 94% of singleton patterns, and over 50 patterns could be found at depth 5 of the *Clinical finding* sub-hierarchy (Fig 6).

By definition, singleton patterns identify SNOMED CT concepts whose structural pattern is unique. Therefore, such SNOMED CT concepts are good candidates for examination to better understand content modeling and potential associated problems. A qualitative assessment was performed for a random sample of fifty SNOMED CT singleton patterns. The sample was stratified by sub-hierarchy and depth, as shown in Table 4.

**Table 4:** Distribution of singleton patterns and assessed stratified sample of 50 concepts.

SNOMED CT Top-level Sub-hierarchy	Depth	Singleton Patterns	Sample
Clinical finding (CF)	2	3	1
	3	4	2
	4	6	2
	5	10	4
	6	14	5
	7	11	4
	8	2	1
	9	2	1
	10	3	1
Event (EV)	2	2	2
Procedure (PR)	1	2	1
	2	2	1
	3	25	2
	4	40	4
	5	54	5
	6	34	3
	7	26	2
	8	5	1
	9	2	1
	10	2	1
Situation with explicit context (SI)	1	1	1
	2	2	1
	3	3	1
Specimen (SN)	1	2	1
	2	3	1
	3	1	1
	**TOTAL**	**261**	**50**

In our assessment, we added the top-level sub-hierarchy of concepts (also known as a semantic tag) in parentheses after the concept name, in agreement with the standard SNOMED CT nomenclature. Note also that we refer to SNOMED CT concepts or singleton patterns indistinctly.

## Procedures

The most obvious observation was that twelve concepts (among which eleven were *Procedures*) out of the 50 randomly selected singleton patterns were highly pre-coordinated, which could be ascertained by multiple (three or more) role groups. In SNOMED CT’s *Procedure* sub-hierarchy, *role groups* [35] stand for subprocedures, as in the concept in Table 5, which describes the removal of an ear ossicle (first subprocedure) with the revision of the ear tympanon (second procedure).

**Table 5:** Stated relationships and role groups for the SNOMED CT concept *Ossiculectomy with tympanoplasty revision (procedure)*.

Attribute	Role group	Target concept with semantic tag
**Is-a**	0	*Revision (procedure)*
**Is-a**	0	*Ossiculectomy of middle ear (procedure)*
**Method**	1	*Excision—action (qualifier value)*
**Procedure site—Direct**	1	*Ear ossicle structure (body structure)*
**Method**	2	*Surgical action (qualifier value)*
**Procedure site**	2	*Tympanic membrane structure (body structure)*
**Revision status**	2	*Revision—value (qualifier value)*

Both entries in role group 1 in Table 5 are already part of the definition of their stated parent, *Ossiculectomy of middle ear (procedure)*. As these relationships are inherited anyway, they are redundant in the stated relationships table. Analyzed separately, these subprocedures instantiate relatively frequent patterns. For example, the combination of the relationships **Method**, **Procedure site**, **Revision status** with the range types *Qualifier value*, *Body structure*, *Qualifier value* occurred 98 times. Although this and other examples do not exhibit inconsistent modeling, they indicate potential problems in content management due to the redundancy of relationships contained in the stated version, in good agreement with Dentler et al. [9].

Patterns for such combined procedures reinforce the tacit ontological assumption that a combined procedure concept is a common taxonomic child of the subprocedures, according to the interpretation of the role group relationship as **hasPart** [36]. In description logics:

*ProcA* equivalentTo *Procedure* and **hasPart** some (**r**_1_ some *t*_1_ and **r**_2_ some *t*_2_ and … **r**_k_ some *t*_k_)

*ProcB* equivalentTo *Procedure* and **hasPart** some (**s**_1_ some *u*_1_ and **s**_2_ some *u*_2_ and … **s**_k_ some *u*_k_)

*ProcAwithB* equivalentTo *ProcA* and *ProcB*

From a maintenance point of view, it would be easier to assure that the simple procedure concepts are sufficiently represented, which would then simplify the representation of the combined procedures as shown in Table 6.

**Table 6:** Alternative, a more parsimonious model for representing *Ossiculectomy with tympanoplasty revision (procedure)*.

Attribute	Role group	Target concept with semantic tag
**Is-a**	0	*Ossiculectomy of middle ear (procedure)*
**Is-a**	0	*Revision of myringoplasty (procedure)*

The current modeling approach (Table 5) requires inferring subclass (**is-a**) relationships by description logics reasoning. The risk is that such relationships are not computed due to debatable modeling decisions made elsewhere. For instance, the second **is-a** relationship in Table 6 is missing in the current inferred version, as the target concept *Revision of myringoplasty (procedure)* exists but is, probably by mistake, not fully defined.

The reason for the uniqueness instantiated by the concept *Catecholamines*, *fractionation measurement*, *urine (procedure)* (Table 7) is the specification of the measurement method, which is not reflected in the name and which contrasts with the definition of similar concepts in which the measurement method is not specified.

**Table 7:** *Catecholamines*, *fractionation measurement*, *urine (procedure)*: Procedure concept definition, missing Specimen, but having a measurement method specified that is not reflected in the name.

Attribute	Role group	Target concept with semantic tag
**Component**	0	*Catecholamine (substance)*
**Has specimen**	0	*24-hour urine sample (specimen)*
**Is-a**	0	*Measurement of substance (procedure)*
**Measurement method**	0	*High pressure liquid chromatography (procedure)*

In other cases for which the measurement method was specified, as in *Urobilinogen concentration*, *test strip measurement (procedure)*, the measurement method was specified (according to the wording of the term), but the *Specimen (urine)* was not specified, which points at another modeling discrepancy. In these and many other similar concepts, we again found a duplication of identical relationship–target pairs between parent and child concepts, thus introducing unnecessary redundancy in the stated relationship table.

## Events

The following two concepts in the sample were taken from the *Event* sub-hierarchy: *Death by fire* and *Death by overwork*. Both have a simple structure, viz. “**Causative agent** *Fire (physical force)*” and “**Associated with** *Overwork (finding)*”, respectively (Table 8).

**Table 8:** Example of different patterns for obviously similar concepts in the Events sub-hierarchy.

Concept defined	Attribute	Role group	Target concept with semantic tag
*Death by fire (event)*	**Is-a**	0	*Death (event)*
	**Causative agent**	0	*Fire (physical force)*
*Death from overwork (event)*	**Is-a**	0	*Death (event)*
	**Associated with**	0	*Overwork (finding)*
*Death by electrocution (event)*	**Is-a**	0	*Death (event)*
	**Due to**	0	*Exposure to electric current*, *with passage of current through tissue (event)*
*Death due to radiotherapy toxicity (event)*	**Is-a**	0	*Death (event)*

Whereas the differences in modeling (**Causative agent** vs. **Associated with**) are consistent with the concept model, which allows different ranges for these two relationships, the modeling differences between *Death by fire* and *Death by electrocution* shows a subtle difference between *physical force* and **Exposure to** a *physical force*. A recommendation could be to substitute all *physical force* concepts *P* to **Exposure to** *P*, and make sure that all needed exposures are present so that, e.g., *Death due to radiation* can be expressed in the same way. Additionally, this analysis suggests implausible choices of the relationship **Associated with** in one case and **Due to** in another case. A revision of the concepts using the semantically shallow relationship **Associated with** has already been suggested by the IHTSDO Event Condition Episode group [37].

## Clinical Findings

With *Clinical finding* (disease) concepts, the reasons for uniqueness were rather diverse. For instance, the concept *Accessory ossification center (disorder)* is linked via **Has interpretation** to *Abnormal (qualifier value)* (Table 9).

**Table 9:** Disease, combined with the qualifier *Abnormal*, as a modeling idiosyncrasy in *Accessory ossification center (disorder)*.

Attribute	Role group	Target concept with semantic tag
**Is-a**	0	*Disease (disorder)*
**Associated morphology**	1	*Supernumerary structure (morphologic abnormality)*
**Finding site**	1	*Bone structure (body structure)*
**Occurrence**	1	*Congenital (qualifier value)*
**Has interpretation**	2	*Abnormal (qualifier value)*
**Interprets**	2	*Bone formation*, *function (observable entity)*

Regardless of other components of the pattern it instantiates, we found that the combination of a disorder with the relationships **Occurrence** and **Has interpretation** yields only this single concept. The meaning of **Has interpretation** is, typically, to characterize a finding related to some parameter that is normal or abnormal. It is counterintuitive to use this for the characterization of disorders, which are, by definition, abnormal. In addition, the combination of the relationships **Associated morphology** and **Interprets** in the same concept definition was very rare. This supports the phenomenon, already discussed in IHTSDO circles, that the use of the relationship **Interprets** is not very principled and has therefore not been used consequently. Because it points to a concept of the type *observable entity* (ontologically to be interpreted as an information entity), it creates in the abovementioned OWL interpretation an ontological dependency of disorders or findings on information artifacts, which is simply wrong, because disorders may exist without being referred to by some piece of information in a health record.

Table 10 shows that the modeling of composed concepts of the type *A* **Due to** *B* follows different patterns. Although the concept *Congenital toxoplasmosis (disorder)* exists, it is neither referred to, nor can it be inferred by reasoning. Instead, several characteristics of this disease are replicated in the complex concept *Lymphadenopathy due to congenital toxoplasmosis (disorder)*.

**Table 10:** *Lymphadenopathy due to congenital toxoplasmosis (disorder)*: *a* complex concept without direct reference to the underlying etiology (*Congenital toxoplasmosis*).

Attribute	Role group	Target concept with semantic tag
**Is-a**	0	*Lymphadenopathy (disorder)*
**Causative agent**	1	*Toxoplasma (organism)*
**Finding site**	1	*Structure of lymph node (body structure)*
**Occurrence**	1	*Congenital (qualifier value)*
**Pathological process**	1	*Parasitic process (qualifier value)*

In the most recent release of SNOMED CT, the concept was redefined following the updated SNOMED CT Editorial Guide by adding the axiom **Due to** *Congenital toxoplasmosis*. The rest of the attributes and values were inactivated.

Table 11 shows another example of modeling weaknesses in complex concepts that are typically found in singleton patterns. As in Table 8, a concept with the tag *physical force* is referred to, where in the same way an event concept **Exposure to** would be justified, which would then require the relationship **Due to**. Furthermore, the reference to exposure would better fit the etiology, viz. *Radiation therapy*, a concept that exists but is not linked to. Finally, again, the second relationship–target pair also holds for the parent *Disorder due to exposure to ionizing radiation (disorder)*, another example of the phenomenon that the set of relational expressions for a concept is unnecessarily blown up by content that is also inherited by a parent concept.

**Table 11:** *Adverse effect of radiation therapy (disorder)*: example of uniqueness, due to non-consequential modeling of concepts that include a reference to their etiology in their definition but not in their formal representation.

Attribute	Role group	Target concept with semantic tag
**Associated with**	0	*Radiation oncology AND/OR radiotherapy (procedure)*
**Causative agent**	0	*Ionizing radiation (physical force)*
**Is-a**	0	*Disorder due to exposure to ionizing radiation (disorder)*

That a given relationship–target constellation is introduced at a high hierarchical level, exactly because it is then inherited by all descendants, may be a fully acceptable explanation for a unique pattern. This was the case for *Procedure with explicit context (situation)* (Table 12).

**Table 12:** *Procedure with explicit context (situation)*: a high-level singleton pattern.

Attribute	Role group	Target concept with semantic tag
**Temporal context**	0	*Temporal context value (qualifier value)*
**Procedure context**	0	*Context values for actions (qualifier value)*
**Is-a**	0	*Situation with explicit context (situation)*

However, the rationale behind this model is not clear. On the one hand, the target concepts are completely unspecific. This could be seen as empty templates to be mandatorily filled by the descendants. On the other hand, the relationship **Associated procedure** *Procedure (procedure)* was not included, although this necessarily characterizes all descendants of *Procedure with explicit context (situation)*.

## Discussion

Most prior work on evaluating SNOMED CT’s modeling problems has focused on clustering problematic concepts into groups for further inspection. While such clusters (e.g., positional similarity sets [13], or areas and p-areas [19,20]) are helpful for quality assurance, they face difficulties when pinpointing the origin of those modeling problems in SNOMED. Our approach, however, analyzes latent patterns among stated relationships in the underlying SNOMED CT model [26], for research has shown that parts of an ontology that do not follow an explicit modeling pattern tend to have more potential defects [8]. In particular, we examined, depthwise and breadthwise, *structural patterns* that reflect directly the way editors model concepts in SNOMED CT.

*Clinical finding* and *Procedure* alone accumulated 92% of the total 852 structural patterns. It is understandable that *Procedure* and *Clinical finding* are two key sub-hierarchies that have been actively modeled and updated. Many factors could contribute to the large number of structural patterns. Various subject areas in a sub-hierarchy require different combination of attributes in the concept model, such as surgical procedures versus diagnostic imaging procedures. The association between the number of patterns and the number of attributes of each sub-hierarchy indicates the potential of increased variations of attribute combination. Different styles of modeling, such as omitting or duplicating attributes and values from parent concepts, could also increase the number of structural patterns, though the differences will not be represented in the inferred version. These identified patterns in the stated version are therefore helpful for analyzing the potential causes of modeling variations.

Because the current version SNOMED CT has not implemented advanced description logics features such as role chain and reflective property characteristic, the depth of a concept in a sub-hierarchy can be mainly based on the combination of relationships, the granularity of subject, and the depth of the object in other sub-hierarchies. Our analysis shows that the number of structural patterns peaked at the depth of level five. Further examination of how concepts were modeled in the *Procedure* and *Clinical finding* sub-hierarchies reveals that the majority of concepts were modeled using fewer than five distinct attributes. Concepts at a depth greater than six could be a result of granularity of subject and depth of object in other hierarchies such as body structure, methods, and morphology. The exhaustion of combinations of attributes could be the cause of reduction in structural pattern numbers towards the deepest concepts.

Our results also show that most patterns were very specific, i.e., they were not reused to model other concepts. This lack of pattern reuse (particularly in the *Procedure* and *Clinical finding* sub-hierarchies) is surprising, as one would expect that editors would benefit from modeling as many as possible using a single structural pattern or template. In addition, we found that structural patterns were unevenly distributed both breadthwise and depthwise, correlating with SNOMED CT’s shape. A future study could include role grouping and assignment of primitive/fully defined super-concepts as additional characteristics for analyzing patterns. This analysis would provide more precision to the existing modeling styles and identify potential improvements for consistent modeling.

The analysis of a random sample of 50 singleton patterns showed a broad range of modeling problems, not only confirming the well-known intra-axiom redundancy between parents and children [9] but also revealing (i) expected inferred **is-a** relationships that were missing in the inferred version, (ii) inconsistencies in secondary disorders, sometimes defined in terms of the primary disorder and sometimes with reference to the pathogen (agent), and (iii) inconsistency in the use of causal links (**Exposure to** and **Due to**).

## Conclusions

In this study, we aimed to improve our understanding of modeling idiosyncrasies in SNOMED CT, a comprehensive clinical health terminology modeled using description logics that constitutes an important blueprint for terminology standardization.

For that purpose, we studied the distribution of *structural patterns* in stated relationships breadthwise and depthwise, as they directly represent the modeling strategies of SNOMED CT editors. We were particularly interested in investigating *singleton patterns*—structural patterns that were only used once—as candidates for revealing modeling problems. Our results showed that the 852 structural patterns found were unevenly distributed as follows: breadthwise, the majority of structural patterns (92%) were accumulated in only two sub-hierarchies (*Procedure* and *Clinical finding*), and depthwise, patterns peaked at depth 5. Similar results were found when analyzing the 261 singleton patterns as follows: 94% of the singleton patterns accumulated under *Procedure* and *Clinical finding* and most were at depth 5.

The broad range of problems detected in a sample of 50 singleton patterns suggests that the analysis of structural patterns is a valuable technique for revealing problematic concepts in SNOMED CT and other comprehensive terminologies described using description logics. The patterns with larger numbers of concepts identified in this study could provide insight for template creation and refinement. In return, the constraints in template-based concept modeling could reduce the variations of modeling decisions. Our study is not limited to addressing quality assessment details for SNOMED CT in particular, but it conveys important lessons for the practice of terminology engineering in general.
